# Prospective Study on the Effectiveness of Complementary Food Supplements on Improving Status of Elder Infants and Young Children in the Areas Affected by Wenchuan Earthquake

**DOI:** 10.1371/journal.pone.0072711

**Published:** 2013-09-09

**Authors:** Caixia Dong, Pengfei Ge, Xiaolan Ren, Jie Wang, Haoqiang Fan, Xiang Yan, Shi-an Yin

**Affiliations:** 1 Department of Chronic Diseases, Gansu Center for Disease Control and Prevention, Lanzhou City, Gansu Province, China; 2 Department of Maternal and Child Nutrition, National Institute of Nutrition and Food Safety, Chinese Center for Disease Control and Prevention, Beijing, China; 3 The Department of Geriatrics, The first Hospital of Lanzhou University, Lanzhou City, Gansu Province, China; Aga Khan University, Pakistan

## Abstract

**Objective:**

To prospectively evaluate the efficiency of daily providing complementary food supplements decreasing malnutrition and anemia prevalence in elder infants and young children living in areas affected by Wenchuan Earthquake.

**Design:**

Using promotional probability sampling method, 250 to 300 children from six-randomized townships (30 to 50 children in each township) in Kang County affected by the Earthquake were randomly chosen for follow up to evaluate intervention effectiveness using anthropometric measurement and hemoglobin level at six, twelve and eighteen months after start of intervention.

**Setting and Subjects:**

All children from 6 to 18 months of age in Kang County (in North Western China) were daily provided with complementary food supplements containing multiple vitamins and minerals for up to 24 months of age. The intervention period lasted for one and half year.

**Results:**

At beginning of intervention, malnutrition prevalence, including underweight, stunting and wasting were respectively 4.5%, 8.9% and 3.5%; anemia prevalence was 74.3%. After one and half year intervention, the growth and anemia status were significantly improved; the percentages of wasting, stunting underweight prevalence were decreased from 3.5%, 8.9% and 4.5% to 1.7%, 5.0% and 3.3% respectively, and the anemia rates were significantly decreased.

**Conclusions:**

Our results indicated that an intervention using complementary food supplements could improve nutritional status and elevate hemoglobin level in elder infants and young children, which would significantly decrease the prevalence of malnutrition and anemia.

## Introduction

On May 12^th^, 2008, a devastating earthquake occurred in Wenchuan County of China, which affected about 348 million people. Sichuan, Gansu and Shan-xi Provinces in China were the worst affected areas. Kang County is located in the southeastern part of Gansu Province and is near the seismic center of the earthquake. The living conditions in this County dramatically have been changed for entire communities, and normal lives were seriously disrupted. Food supply systems were severely damaged or even completely stopped, and major food shortages became a primary post-earthquake feature.

In such emergency, past experience has shown that even in previously healthy population, infant and young children's morbidity and mortality often dramatically increased in a very short period [1], since infants and young children are the most nutritionally vulnerable group after a natural disaster [2]–[5]. Micronutrient deficiencies were reported to be very common in infants and young children in China [5], [6] as well as many developing countries [7], [8] and often exacerbated by a general deterioration in macronutrient supply [5]. Based on our monitoring data on children under 60 months of age one year after the earthquake in this County [5], the prevalence of anemia of 0- months old children and 24–59 months old children were 47.5% and 21.5%, respectively; the prevalence of iron and zinc deficiencies were 45.7%, and 65.5%; the prevalence of stunting was 13.6% among the 24–59 months old children.

The period from 6 to 24 months of age is the most critical for a young child because of their rapid growth and an increasing reliance on complementary food. In order to improve nutritional status and reduce the mortality of infants and young children, adequate food-based nutrition interventions should be carried out, since such interventions might play a key role in saving lives through their impact on the nutrition and health of the target population aged 6 to 24 months of age. In the developing countries, food-based interventions have been demonstrated to be an efficiency way to decrease malnutrition and iron-deficient anemia among the children aged 6–59 months [9]–[13]. Such as iron-fortified milk and noodle or biscuits [9]–[11], and multiple micronutrient-fortified powder [12], [13] have been used for intervention to improve growth and other outcomes in young children. However, the effectiveness on using complementary feeding supplements fortified with multiple micronutrients for infants and young children have not been throughout evaluated in the poor areas or disaster areas.

In order to improve the nutritional status of infants and young children in affected areas, an intervention using complementary food supplements was carried out in Kang County from May 2010 to October 2011. This intervention covered all infants and young children aged 6–24 months of age in these areas. This study was supported by the United Nations International Children's Emergency Fund (UNICEF) and organized by the National Institute of Nutrition and Food Safety, Chinese Center for Disease Control and Prevention (NINFS-CCDC). This paper focuses on the intervention efficiency of complementary food supplements on decreasing malnutrition and anemia prevalence of children from 6 months to 24 months living in disaster areas.

## Materials and Methods

Kang County, located in high mountain areas, has often been attacked by natural disasters in history. This County belongs to a hardest hit area and life and property losses of local residents were very severe. This county is categorized by the Chinese government as poverty-stricken county, and consists of 20 townships and more than 200 000 residents.

### Subjects

After two years of earthquake, a complementary food supplement trial was conducted across rural sites in all Kang County with baseline levels of high stunting (8.9%%) and anemia prevalence (74.3%) in infants and young children. All resident children from 6 to 18 months of age in villages were daily provided with complementary food supplements (Referred to as “Yingyang Bao” in Chinese) containing multiple vitamins and minerals. This intervention project was approved by the ethic committee of NINFS-CCDC, and written informed consent was obtained from all parents or guardians before enrolling children.

### Yingyang Bao distribution

Yingyang Bao was purchased through public competitive bidding by UNICEF in China, and the product of complementary food supplements produced by Qingdao Biomate Food Company, LTD in China was selected for use in this study. At every three months, Yingyang Bao was distributed to the County Center for Disease Control and Prevention (CDC), and then local CDC distributed these products to the township clinics and to the villages. This intervention lasted for one and half years (May 2010∼October 2011), Yingyang Bao was distributed to all children aged from 6 to 18 months at the beginning of the project until children reached 24 months of age. The nutritional status data were collected at the baseline, six months, and twelve months and at eighteen months (the end). The nutrition quality and safety were evaluated and monitored by NINFS-CCDC and the third Laboratory (Intertek Group plc and SGS in China) designed by UNICEF.

This product was specially designed for the elder infants and young children based on a China's National Standard for “General standard for complementary food supplements (GB/T 22570-2008). Ingredients of formulated supplementary foods were as follows (Nutrients/10 g/pack/d): protein 3.0 g from soybean, vitamin A 250 µg, vitamin D_3_ 200 IU (5 µg), vitamin B_1_ 0.3 mg, vitamin B_2_ 0.3 mg, iron 5 mg, zinc 5 mg and calcium 250 mg. In addition, one pack contained energy 40 kcal, fat 2.0 g and carbohydrates 2.5 g.

### Compliance evaluation

Staffs designated in each village, township, and county were responsible for filling in the forms of every monthly distribution records of Yingyang Bao, collected the number of local children from 6 to 24 months of age, checked monthly household taking Yingyang Bao table and uploaded these data to Provincial CDC and National CDC for calculating the compliance. The proportion of children taking Yingyang Bao more than 4 times per week accounted for more than 90% calculated by our every monthly distribution records.

### Monitoring sample size and time

Using promotional probability sampling (PPS) methods, total of 250 to 300 children from six-randomized townships sampling from 20 townships were randomly chosen for follow up to evaluate intervention effectiveness at six months, twelve months and eighteen months after start of intervention on May 2010, respectively. In each township, sample size consisted of 30 to 50 children.

At baseline survey, the number of children investigated in each sampling township and the percentage accounted for local children were as follow 43 in Chengguan (14.1%), 59 in Pingluo (19.5%), 39 in Anmenkou (12.9%), 41 in Yangba (13.6%), 53 in Wangba (17.6%), and 67 in Douba (22.2%); the percentages of children were 33.8% in the group from 6 to 11 months, 37.1% in the group from 12–17 months, and 29.1% in the group from 18–24 months, respectively.

### Bias

Based on the study design and ethic consideration, we could not have a control group during our intervention study in disaster areas. This intervention study is a pre-post design with no control group. Because Kang County located in the high mountain areas and the living place was much decentralized and inaccessible areas, the sample size at each monitoring point was not inconsistent by the reasons that some of the children could not catch up with the measurement due to the bad weather and raining, or inaccessible transportation from their living areas, which could lead to the potential bias.

Because the baby reached to 6 -month-old continually entered to 6∼12 month group and some children from this group transferred into 12∼18 month group, such continuing change could also lead to significant bias in intervention efficiency of the 6–12 month group.

### Methods

A questionnaire was answered by child's mother or other caregiver at each monitoring time point. The body weight, length and hemoglobin (Hb) concentration were measured to evaluate the growth status, malnutrition and anemia prevalence.

A platform weighing scale (TC100KA, 0–100 kg of capacity and 10 g of accuracy, Huatec company, China) and length scale (YSC-2, 0.1 cm of accuracy, Beijing Guowangxingda weight Scale Company, China) were used to measure body weight and length of children wearing only underwear. Before each measurement, the weighing and length scales were checked with the calibrated weight and length materials. The evaluating nurses should had been trained and evaluated for accuracy and consistency of results.

Hb concentration of whole blood, taken from the tips of left middle finger, was assayed using the hemocue (HB 301, HemoCue AB, Angelholm, Sweden) in the field sites. Anemia was defined as a Hb level of <110 g/L, for those sites with an altitude over 1000 meters, the anemia prevalence was corrected using the altitude formula based on Hb concentration was increased by 4 g/L per 1000 m when altitude was over 1000 m [14]. To determine the level of geographical clustering of blood Hb concentration, we used altitude-adjusted Hb concentration values in children <5 years of age (data available from corresponding author).

### Statistical analysis

Epidata software (Windows version 3.1, Epidata, Denmark) was used to enter and manage the raw data. SAS software (version 9.1; SAS Institute Inc, Cary, NC) was used for the statistical analysis. Main outcome measures included principal determinants of undernutrition and childhood stunting, wasting and underweight. WHO Anthro software was used to calculate the children growth status, the means of body length and weight, length-for-age z score (LAZ), weight-for-age z score (WAZ), and weight-for-length z score (WLZ). Growth status was evaluated by WHO Child Growth Standard (2006), and malnutrition was defined as a z score less than -2. Data were expressed as mean±SD.

## Results

### Basic characteristics

Evaluation of intervention efficiency and Yingyang Bao distribution were respectively carried out at the baseline, six months, twelve months, and eighteen months by trained staff from the Center for Disease Control and Prevention at the County, Provincial and National levels. Evaluation surveys were carried out at the 4 time points and we assayed 1019 children aged 6–24 months of age including 314, 227, 236, and 242 infants and young children from baseline to the end of intervention. Compliance taking Yingyang Bao was more than 90% calculated based on the statistic data provided by villages, townships and county. The percentage of exclusive breastfeeding of infants under 6-months was about 59%. There were only 11% infants and young children aged 0–23 months who could have breastfeeding within 1 hour after delivery. More than 90% of children from 0–23 months old never received any nutritional supplements. The length and body weight of each group at the basal line were shown in Table 1. The length and body weight of boys trended to be higher than that of girls in each age groups.

**Table 1:** The body weight (kg) and length (cm) of each group at the basal line.

Age	Total			Boys			Girls		
(m)	n	Length	weight	n	Length	weight	n	Length	Weight
6∼	112	69.2±3.6	8.3±1.2	58	70.4±2.6	8.7±1.2	54	67.8±4.0	7.9±1.1
12∼	89	76.4±3.3	9.6±1.2	40	77.9±3.1	10.1±1.0	49	75.2±2.9	9.2±1.1
18∼24	112	80.7±4.2	10.2±1.2	56	82.0±3.9	10.7±1.2	55	79.3±4.1	9.7±1.0

The results were expressed as Mean±SD.

### The prevalence and extent of malnutrition and anemia at baseline

**Figure 1:**
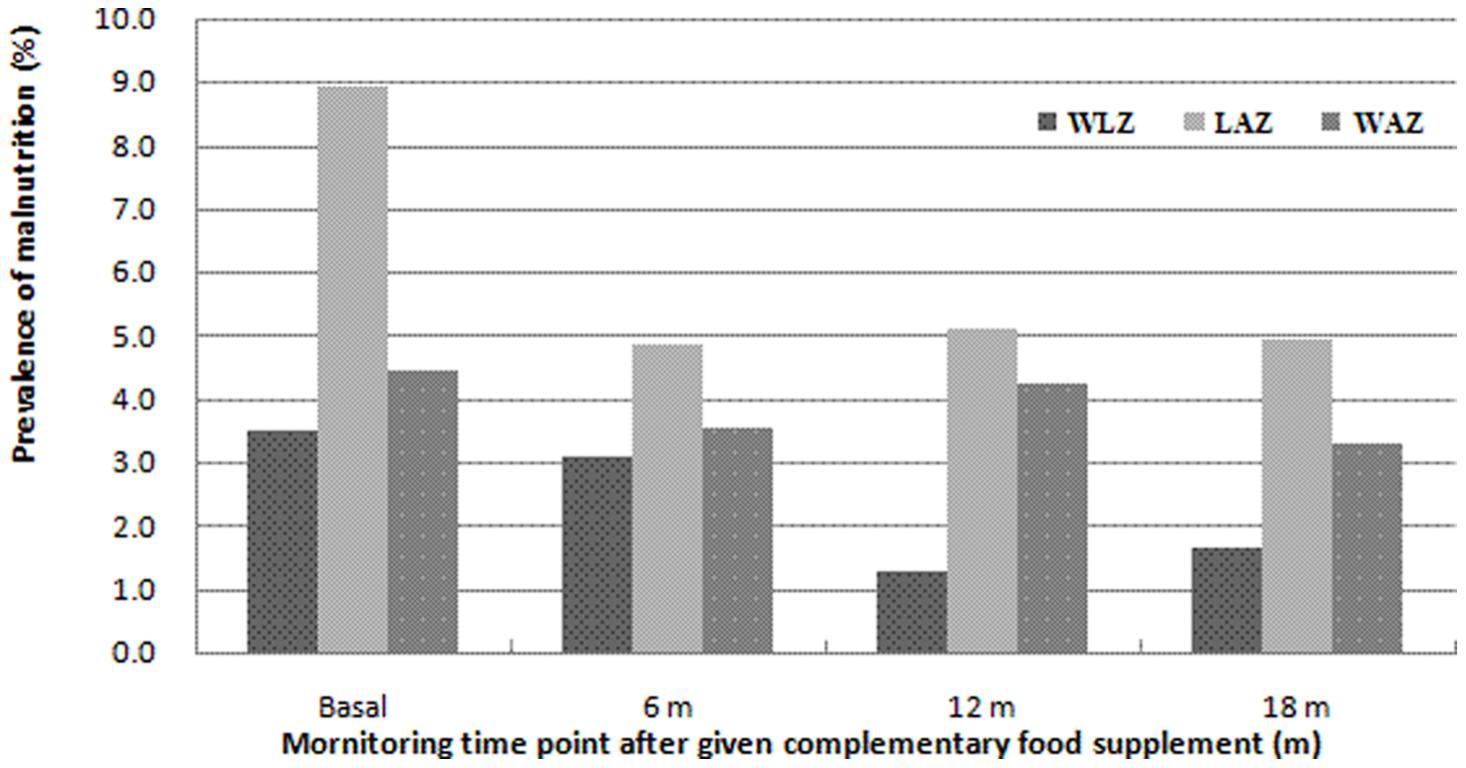
The effect of given the complementary food supplements on prevalence of malnutrition. Wasting (WLZ←2), stunting (LAZ←2) and underweight (WAZ←2).

**Figure 2:**
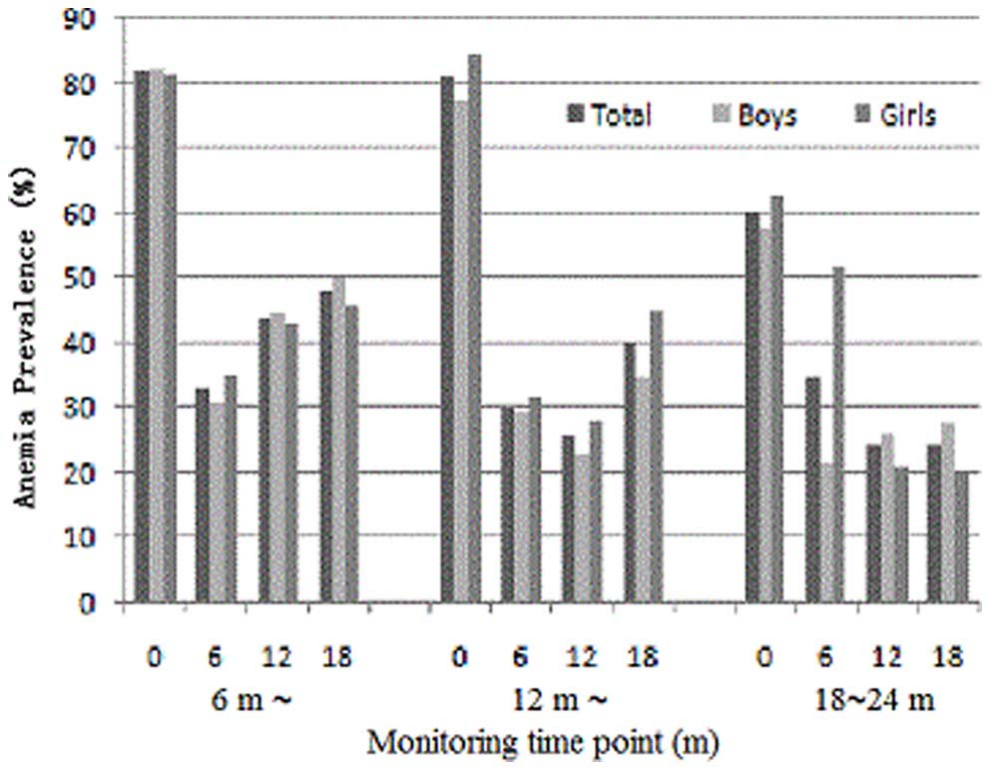
The effect of complementary food supplements on prevalence of anemia in the groups aged 6 months∼, 12 months ∼, and 12∼24 months.

The extent of malnutrition at baseline was evaluated using the Z-score less than -2SD, including underweight with WAZ<2SD, stunting with LAZ<2SD and wasting with WLZ<2SD, and anemia was diagnosed by the Hb level in whole blood. The prevalence of malnutrition and anemia of infants and young children were shown in Figure 1 and Figure 2 after two years of the earthquake. At beginning of intervention, the baseline monitoring data showed that malnutrition prevalence, including underweight, stunting and wasting was respectively 4.5%, 8.9% and 3.5%. Malnutrition prevalence including underweight and stunting trended increase from 6 to 24 months. Anemia prevalence was 74.3%. Based on malnutrition and anemia prevalence, the nutrition status of infants and young children was worsening in these areas.

### The intervention efficiency of complementary food supplements on child's growth and hemoglobin level

The improving efficiency of complementary food supplements on length-for-age z score (LAZ), weight-for-age z score (WAZ), and weight-for-length z score (WLZ) was shown in Table 2. After introducing the complementary food supplements, the trend of growth and development status was significantly improved in different age groups and there was no significant difference between boys and girls.

**Table 2:** The change of Z-score of infants and young children at different monitoring time point after given complementary food supplements.

Monitoring	Total				Boys				Girls			
time point	n	WLZ	LAZ	WAZ	n	WLZ	LAZ	WAZ	n	WLZ	LAZ	WAZ
Infants aged 6–12 months												
Baseline	114	0.23±1.14^ab^	−0.46±0.09^a^	−0.09±1.11^a^	58	0.22±1.26^a^	−0.48±0.98^a^	−0.11±1.21^a^	56	0.24±1.00^ab^	−0.44±0.88^a^	−0.06±1.00^ab^
6 month	65	0.06±0.93^a^	−0.08±1.15^b^	−0.05±1.01^ab^	29	0.04±1.08^a^	0.07±1.17^b^	0.03±1.08^ab^	36	0.07±0.81^a^	−0.19±1.13^a^	−0.12±0.96^b^
12 months	68	0.36±0.90^b^	−0.25±1.31^ab^	0.10±1.19^b^	39	0.26±1.02^a^	−0.42±1.36^ab^	−0.12±1.31^ab^	29	0.49±0.72^b^	0.00±1.22^a^	0.38±0.96^c^
18 months	58	0.44±0.87^b^	−0.04±1.06^b^	0.32±0.95^c^	34	0.47±1.04^a^	0.04±1.26^b^	0.41±1.17^b^	24	0.40±0.55^ab^	−0.16±0.71^a^	0.19±0.47^abc^
Young children aged 12–18 months												
Baseline	111	−0.08±0.96^abc^	−0.35±1.08^a^	−0.22±0.98^abc^	53	−0.12±0.96^ab^	−0.23±1.17^a^	−0.21±0.96^a^	58	−0.04±0.97^ab^	−0.45±1.00^ab^	−0.24±1.00^abc^
6 month	87	−0.36±1.01^ab^	−0.25±1.19^a^	−0.38±0.97^ab^	47	−0.32±1.03^a^	−0.30±1.02^a^	−0.38±1.04^a^	40	−0.40±1.01^a^	−0.20±1.38^ab^	−0.37±0.88^ac^
12 months	89	−0.18±0.97^b^	−0.51±1.01^a^	−0.36±0.96^b^	39	−0.11±1.09^ab^	−0.41±0.97^a^	−0.26±1.00^a^	50	−0.23±0.86^ab^	−0.59±1.05^a^	−0.44±0.93^c^
18 months	92	0.17±0.90^c^	−0.26±1.40^a^	0.00±1.00^c^	43	0.25±0.92^b^	−0.47±1.60^a^	−0.04±1.10 a	49	0.10±0.89^b^	−0.08±1.18^b^	0.04±0.92^b^
Young children aged 18–24 months												
Baseline	89	−0.34±0.99^a^	−1.04±1.20^a^	−0.76±0.86^a^	43	−0.17±1.02^a^	−0.79±1.09^a^	−0.50±0.83^a^	46	−0.50±0.95^a^	−1.28±1.27^a^	−1.00±0.84^a^
6 month	75	−0.33±0.93^ab^	−0.57±1.11^b^	−0.52±0.86^ab^	45	−0.43±0.88^a^	−0.52±1.06^a^	−0.58±0.79^a^	30	−0.18±1.01^ab^	−0.64±1.19^b^	−0.43±0.95^b^
12 months	79	−0.04±0.88^a^	−0.70±0.85^b^	−0.37±0.89^b^	46	−0.15±0.85^a^	−0.63±0.85^a^	−0.42±0.84^a^	33	0.11±0.93^b^	−0.79±0.85^b^	−0.30±0.95^b^
18 months	92	−0.09±1.04^ab^	−0.47±1.27^b^	−0.28±0.99^b^	46	−0.08±1.21^a^	−0.45±1.42^a^	−0.28±1.11^a^	46	−0.10±0.84^b^	−0.48±1.10^b^	−0.29±0.85^b^

The results were expressed as means ± SD. Means in the same age group within same column with different superscripts a–c were significantly different as a result of intervention at the *P<0.05* level.

The Hb concentration (g/L) of infants and young children was shown in Table 3. After introduction of complementary food supplements from six months of age, the average Hb level was significantly increased in each age group, and this trend remained stable up to the end of supplementation, and there was no significant difference between boys and girls.

**Table 3:** Hemoglobin concentration change of infants and young children at different monitoring time point after given the complementary food supplements (g/L).

Monitoring time point	Total		Boys		Girls	
	n	Average	n	Average	n	Average
Infants aged 6–12 months						
Baseline	114	104.4±12.6^a^	58	103.1±1.36^a^	56	105.8±11.4^a^
6 month	66	118.5±12.6^b^	29	120.7±11.4^b^	37	116.8±13.3^b^
12 months	68	114.3±9.4^c^	39	113.7±7.0^c^	29	115.1±11.9^b^
18 months	58	113.3±14.0^c^	34	110.9±13.4^c^	24	116.6±14.4^b^
Young children aged 12–18 months						
Baseline	111	104.7±12.7^a^	53	107.8±12.0^a^	58	101.8±12.8^a^
6 month	87	119.0±12.9^b^	47	118.6±13.0^b^	40	119.5±12.9^b^
12 months	89	118.0±9.3^b^	39	118.4±9.9^b^	50	117.6±8.8^bc^
18 months	92	115.2±9.0^c^	43	116.0±9.0^b^	49	114.6±9.0^c^
Young children aged 18–24 months						
Baseline	89	110.3±12.1^a^	43	113.4±12.7^a^	46	107.4±10.8^a^
6 month	75	119.3±14.8^b^	45	121.4±14.6^b^	30	116.2±14.7^b^
12 months	79	119.4±9.3^b^	46	118.3±9.0^b^	33	120.8±9.5^b^
18 months	92	120.5±11.9^b^	45	119.2±11.5^b^	46	121.7±12.3^b^

The results were expressed as means ± SD. Means in the same age group within same column with different superscripts a–c were significantly different as a result of intervention at the *P<0.05* level.

### The evaluation on improving efficiency of complementary food supplements on malnutrition and anemia

The effects of introducing complementary food supplements on prevalence of wasting (WLZ←2), stunting (LAZ←2) and underweight (WAZ←2) were shown in Figure 1. The percentages of wasting and stunting prevalence decreased from 3.5% and 8.9% to 1.7% and 5.0% respectively, and underweight rate declined from 4.5% to 3.3%.

The impact of complementary food supplements on anemia prevalence was shown in Figure 2, which showed the trend of anemia prevalence of different age group at monitoring time point (baseline, half year, one year, and one and half year after starting intervention). There was much higher anemia prevalence in each age group at baseline. Through the intervention using complementary food supplements, the anemia rates were significantly decreased in boys and girls, manifested by significantly decreased anemia prevalence from 74.3% at baseline level to 37.4% after six month nutritional intervention. The higher prevalence in the group aged 6–12 months at the end of intervention was related to this group with continued some new entrants reaching 6 months of age and some children from this group entered into 12–18 month group, which indicated that significant bias in intervention efficiency of the 6–12 month group was associated with these changes.

## Discussion

The adverse effects of chronic, acute malnutrition and micronutrient deficiencies on growth and development are very profound in infants and young children [2], [5], [15]. Malnutrition and micronutrient deficiency and inappropriate feeding practices during the first two years of life could lead to increase mortality, morbidity and susceptibility to infection, blindness, adversely affect child's growth and development, and has an irreversible long-term negative impact, which all could influence the health and risk suffered from non-communicable diseases at adulthood [15]–[17], [18].

### Malnutrition and anemia were a serious problem in infants

The reasons of malnutrition in early childhood are rather complex with a variety of direct and underlying contributors related to lack of food in quantity or quality, including insufficient breastfeeding period and inadequate introduction of complementary foods, and nutrient loss resulted from some infectious diseases. During emergency such as earthquake, infant and young children would be the most nutritionally vulnerable group [2]–[5]. The children's nutritional status has been reported to be continuously worsening in those areas affected by the earthquake in Wenchuan [2], [4], [5]. Based on our baseline monitoring results on infant and young child's anthropometry two years after the Earthquake, the growth and development status including underweight, stunting and anemia prevalence were gradually worse which indicated the occurrence of acute malnutrition [2], [4], [5]. Body weight of infants and young children from 6 to 24 months was first affected by the food shortage during such emergency. After two years of the earthquake, the percentage of underweight was 4.5% and height gain were also affected (wasting prevalence was 3.5%); however, the wasting rate did not show a further reduction due to the decrease in body length (stunting prevalence was up to 8.9%). The main reasons on the deteriorating nutritional status was the unavailability of complementary foods for elder infants and young children [2], [5], and poor breastfeeding practices, particularly inadequate duration of exclusive breastfeeding in these areas [5].

Iron deficient anemia remains a common problem and 90% of all types of anemia are due to iron deficiency in China [6] which could increase mortality among children less than five years of age. Emergency such earthquake could make this situation even worse [2], [4], [5]. Infants and young children from 6 to 24 months of age appear to be the most vulnerable to iron-deficiency and iron-deficient anemia [6], [19]. There is a significant amount of scientific literature suggests that iron deficiency and iron-deficient anemia may be associated with an impaired immune system [20] which could increase the morbidity and mortality, and with the delay in cognitive function development [21] or behavior problems [22]. The monitoring results from affected sites in our study showed that the anemia prevalence significantly increased in infants and young children from two months to two years of the earthquake, and this negative effect on girls was more significantly, for example, anemia percentage was respectively 76.2% for girls and 72.3% for boys after two years of earthquake. What are the reasons on the high prevalence of anemia at baseline? The great concerns were the inadequacy of iron intake and most of iron from plant foods (non-heme iron), because non-heme iron is relatively unavailable to the absorptive cells, but this kind of iron could constitute a significant amount of iron intake in such areas [4], [6]. Increase of anemia prevalence were also associated with a shortage of high quality foods including lack of animal products, dairy and legume products in their daily diets.

If these children living in such areas could not be quickly given with an adequate nutrition intervention, it would result in increased wasting, more underweight, and greater levels of stunting and wasting, and a higher prevalence of anemia, which could result in the increase of mortality [1], [3]. After the earthquake, the government provided food supplies (a general food basket) for six months which could meet the basic energy and nutrient requirements for adults. The mean per capita cereals (including rice or wheat powder) supply was 500 grams per day after the earthquake, which could provide about 1730 kcal/per day, and if added additional energy from the non-staple food consumption (vegetables and fruits), a general food basket could provide about 2100 kcal energy per person per day that could meet the needs for the basic energy, protein, but fat and micronutrient requirements could not be met. However, for a special physiological state, such as pregnant women, nursing mothers, and infants and young children, these foods could be difficult to meet the needs to a variety of micronutrients so that these populations would be at high risk suffered from vitamin and mineral deficiencies [2], [5], [23]. Our present study supports that increasing food supplies and improving food quality to infants and young children are an urgent public health need in those areas struck by natural disaster.

### The intervention using complementary food supplements could improve nutritional status and decrease anemia prevalence

During infants and young children, it has been reported that growth retardation occurs mainly in the 6–23 months of age, if nutrient requirements are not met, this inhibition on child's height growth would be irreversible [24]. If anemia occurs, the impact on mental and physical are also irreversible [25]. In order to meet the physiological needs of infants and young children aged 6–23 months, the supply of high – quality complementary food to these children can be very important in addition to breast feeding. Food-based fortification ways have been world widely taken to improve the quality of complementary food for infants and young children and to keep improving child's growth and development [26], [27].

The complementary food supplements contain most nutrients which are priority and essential to infants and young children [28]. Intervention using complementary food supplements would be very useful for attaining proper nutrition, health, and development of infants and young children. The present intervention results showed that such intervention really decreased the percentages of wasting and stunting prevalence (from 3.5% and 8.9% to 1.7% and 5.0% respectively), underweight rate (from 4.5% to 3.3%).

The elder infants and young children could be at higher risk susceptible to iron-deficient anemia. If foods rich in iron or supplements containing iron could not be provided, these vulnerable groups are easily suffered from iron-deficient anemia due to the depletion of iron reserves in the body, reduction of iron level in breast milk and lack of iron in their regular complementary foods [29]. Our baseline survey showed that there were 74.3% of elder infants and young children suffered from anemia. Based on our data at the monitoring time point at the six months, anemia prevalence was significantly decreased to near 30% through daily supplementation of complementary food supplements contained iron 5 mg/g, and this beneficial effect lasted during 18 month intervention, which indicated that food-based intervention would be an effective way to reduce anemia in children from 6 to 24 months of age [30], [31].

### Limitations

The results reported here have certain limitations. Based on the ethic consideration, we could not have a control group during such intervention so that this intervention was a pre-post design without control group. We tried to use the children with poor compliance less than 4 times per week as control group compared with the children with good compliance more than 4 times per week, however, the poor compliance children were not suitable as the control group due the limited number (less than 10%) and these children had still received a certain amount of this supplement less than 4 times per week. Because our current study is an observational study, probably the results can not directly demonstrate a causal link. However, such as complementary food supplements have been demonstrated effectively to improve nutritional status and elevate hemoglobin level in elder infants and young children [29]–[31].

In summary, after emergency, infants and young children are vulnerable to malnutrition and anemia; during the recovery phase, the food supplies in quantity and quality for children were not significantly improved in these mountain areas. Intervention using complementary food supplements with multi-micronutrients can decrease the prevalence of malnutrition and anemia.
